# Supplementary material for: Profoundly Disturbed Lipoproteins in Cirrhotic Patients: Role of Lipoprotein-Z, a Hepatotoxic LDL-like Lipoprotein
Source: J Clin Med. 2022 Feb 24;11(5):1223. doi: 10.3390/jcm11051223 (PMC8910943; doi:10.3390/jcm11051223)
Supplement: Supplementary file 1 [file jcm-11-01223-s001.zip › jcm-1568403-supplementary.pdf]

Supplemental Tables

Supplemental Table S1. Clinical characteristics and lipid profile of the PREVEND study population.	
	**N = 6,027**
**Baseline characteristics**	
Sex: men / women, *n (%)*	2,971 (49.3) / 3,056 (50.7)
Age (years), *median (IQR)*	52.5 (43.9-63.1)
BMI (kg/m^2), *median (IQR)*	26.2 (23.7-29.0)
BMI<br>• Normal; ≤ 25 kg/m^2, *n (%)*<br>• Overweight; 25-30 kg/m^2, *n (%)*<br>• Obese; ≥ 30 kg/m^2, *n (%)*	 2,325 (38.6)<br>2,535 (42.1)<br>1,167 (19.4)
Smoking, *n (%)*	1,665 (27.6)
History of cardiovascular disease, *n (%)*	379 (6.3)
History of diabetes, *n (%)*	376 (6.2)
Glucose lowering drugs, *n (%)*	221 (3.7)
Lipid lowering drugs, *n (%)*	576 (9.6)
**Blood tests**	
ALT (U/L), *median (IQR)*	17.0 (13.0-24.0)
AST (U/L), *median (IQR)*	22.0 (19.0-26.0)
GGT (U/L), *median (IQR)*	24.0 (16.0-38.0)
ALP (U/L), *mean ± SD*	66.0 (55.0-79.0)
Bilirubin total, *median (IQR)*	7.0 (5.0-9.0)
Albumin (g/L), *median (IQR)*	44.0 (42.0-45.0)
Fasting glucose (mmol/L), *median (IQR)*	4.8 (4.4-5.3)
**Lipids and lipoproteins**	
Total cholesterol (mmol/L), *median (IQR)*	5.4 (4.7-6.1)
Non-HDL cholesterol (mmol/L), *median (IQR)*	4.1 (3.4-4.8)
HDL cholesterol (mmol/L), *median (IQR)*	1.2 (1.0-1.4)
LDL cholesterol (mmol/L), *median (IQR)*	3.5 (2.9-4.2)
Triglycerides (mmol/L), *median (IQR)*	1.1 (0.8-1.6)

ApoB (mg/dL), *median (IQR)*	89.4 (74.1-105.8)
ApoA-I (mg/dL), *median (IQR)*	128.9 (114.3-145.0)
TRLP (nmol/L), *median (IQR)*	149.5 (110.9-192.9)
Very large TRLP (nmol/L), *median (IQR)*	0.0 (0.0-0.1)
Large TRLP (nmol/L), *median (IQR)*	2.0 (0.3-5.6)
Medium TRLP (nmol/L), *median (IQR)*	11.9 (5.6-22.7)
Small TRLP (nmol/L), *median (IQR)*	46.9 (25.0-79.5)
Very small TRLP (nmol/L), *median (IQR)*	70.6 (37.4-110.1)
TRL size (nm), *median (IQR)*	44.3 (39.8-50.5)
LDLP (nmol/L), *median (IQR)*	1488.7 (1238.2-1746.4)
Large LDLP (nmol/L), *median (IQR)*	362.7 (173.3-553.5)
Medium LDLP (nmol/L), *median (IQR)*	372.1 (114.7-721.4)
Small LDLP (nmol/L), *median (IQR)*	564.5 (324.9-904.8)
LDL size (nm), *median (IQR)*	21.2 (20.8-21.4)
Total HDLP (µmol/L), *median (IQR)*	21.0 (19.3-22.8)
Large HDLP (µmol/L), *median (IQR)*	1.5 (0.8-2.5)
Medium HDLP (µmol/L), *median (IQR)*	5.0 (3.6-6.5)
Small HDLP (µmol/L), *median (IQR)*	14.2 (12.2-16.1)
HDL size (nm), *median (IQR)*	8.9 (8.6-9.3)
HDL subspecies	
• H1P, (µmol/L), *median (IQR)*	3.4 (2.2-4.6)
• H2P, (µmol/L), *median (IQR)*	10.6 (9.0-12.2)
• H3P, (µmol/L), *median (IQR)*	3.2 (2.0-4.4)
• H4P, (µmol/L), *median (IQR)*	1.7 (1.1-2.4)
• H5P, (µmol/L), *median (IQR)*	0.3 (0.1-0.6)
• H6P, (µmol/L), *median (IQR)*	0.6 (0.3-1.4)
• H7P, (µmol/L), *median (IQR)*	0.3 (0.1-0.6)

Data are given in number with percentages (%) or median with interquartile ranges (IQR). Abbreviations: ALP, alkaline phosphatase; ALT, aminotransferase; ApoA, apolipoprotein A; ApoB, apolipoprotein B; AST, aspartate aminotransferase; BMI, body mass index; GGT, gamma-glutamyltransferase; HDL, high density lipoproteins; HDLP, high density lipoprotein particles; LDL, low density lipoproteins; LDLP, low density lipoprotein particles; TRL, triglyceride-rich lipoproteins; TRLP, triglyceride-rich lipoprotein particles.

Supplemental Table S2. Multivariable logistic regression analyses demonstrating independent associations of the presence of LP-Z in 130 pre-transplant cirrhotic patients from TransplantLines

	**Model 1**			**Model 2**			**Model 3**			**Model 4**		
	**OR**	**95% CI**	***P*-value**	**OR**	**95% CI**	***P*-value**	**OR**	**95% CI**	***P*-value**	**OR**	**95% CI**	***P*-value**
Age (years)	0.998	0.954-1.043	0.916	1.010	0.961-1.061	0.698	1.009	0.960-1.061	0.721	1.011	0.955-1.069	0.717
Sex (women *vs.* men)	0.901	0.383-2.119	0.812	0.770	0.295-2.012	0.594	0.739	0.281-1.944	0.540	0.722	0.242-2.151	0.558
Child Pugh classification (A/B/C)	4.681	2.355-9.304	<0.001				1.708	0.733-3.981	0.215	1.352	0.539-3.389	0.520
MELD score				1.406	1.240-1.594	<0.001	1.361	1.189-1.559	<0.001	1.401	1.198-1.638	<0.001
Use of glucose lowering drugs (yes/no)										2.821	0.728-10.926	0.133
Use of lipid lowering drugs (yes/no)										0.511	0.100-2.614	0.420

OR: odds ratio. CI 95%: 95% confidence interval. All models are mutually adjusted for the variables included in the analyses. Abbreviations: MELD score, model for end-stage liver disease score.

Supplemental Table S3. Multivariable linear regression analyses demonstrating independent associations of LP-Z in 130 pre-transplant cirrhotic patients from TransplantLines

	Model 1		Model 2		Model 3		Model 4	
	β	*P*-value	β	*P*-value	β	*P*-value	β	*P*-value
Age (years)	0.998	0.916	-0.147	0.066	-0.147	0.060	-0.139	0.80
Sex (women *vs.* men)	0.901	0.812	0.045	0.563	0.052	0.494	0.039	0.620
Child Pugh classification (continuous)	0.198	0.022			-0.273	0.016	-0.280	0.014
• A *vs.* B and C	-.0.048	0.586			0.263	0.004		
• B *vs.* A and C	-0.202	0.019			-0.181	0.019		
• C *vs.* A and B	0.263	0.002			0.046	0.611		
MELD score			0.452	<0.001	0.653	<0.001	0.660	<0.001
Primary liver disease								
• Storage disease *vs.* other aetiologies							-0.062	0.421
• Autoimmune hepatitis *vs.* other aetiologies							-0.101	0.215
• Cholestatic liver disease (PSC/PBC) *vs.* other aetiologies							0.231	0.003
• Viral *vs.* other aetiologies							0.015	0.853
• Alcohol *vs.* other aetiologies							-0.112	0.159
• MAFLD *vs.* other aetiologies							-0.037	0.634
• Vascular *vs.* other aetiologies							-.0.078	0.310
• Malignancy *vs.* other aetiologies							N.A.	N.A.
• Other group *vs.* other aetiologies							0.037	0.632

β: standardized coefficients. All models are mutually adjusted for the variables included in the analyses. Abbreviations: MAFLD, metabolic associated fatty liver disease; MELD score, model for end-stage liver disease score; PBC, primary biliary cholangitis; PSC, primary sclerosing cholangitis.

**Supplemental Table S4. Linear regression analyses demonstrating independent associations of TRLP, LDLP and HDLP in 130 pre-transplant cirrhotic patients from TransplantLines**						
	**Model 1**		**Model 2**		**Model 3**	
	**β**	***P*-value**	**β**	***P*-value**	**β**	***P*-value**
**TRLP**						
Age (years)	-0.261	0.004	-0.247	0.005	-0.257	0.005
Sex (women *vs.* men)	-0.156	0.073	-0.152	0.079	-0.153	0.078
MELD score	-0.095	0.281			-0.050	0.644
Presence of LP-Z (yes/no)			-0.105	0.223	-0.077	0.465
**LDLP**						
Age (years)	-0.291	0.001	-0.297	<0.001	-0.323	<0.001
Sex (women *vs.* men)	-0.004	0.959	-0.020	0.785	-0.023	0.752
MELD score	0.192	0.025			-0.137	0.133
Presence of LP-Z (yes/no)			0.471	<0.001	0.558	<0.001
**HDLP**						
Age (years)	0.014	0.833	0.126	0.089	0.029	0.661
Sex (women *vs.* men)	-0.097	0.148	-0.077	0.296	-0.088	0.171
MELD score	-0.658	<0.001			-0.508	<0.001
Presence of LP-Z (yes/no)			-0.541	<0.001	-0.256	0.001
β: standardized coefficients. All models are mutually adjusted for the variables included in the analyses. Abbreviations: MELD score, model for end-stage liver disease score; TRLP, triglyceride-rich lipoprotein particles; LDLP, low density lipoprotein particles; HDLP, high density lipoprotein particles.						

Supplemental Figures

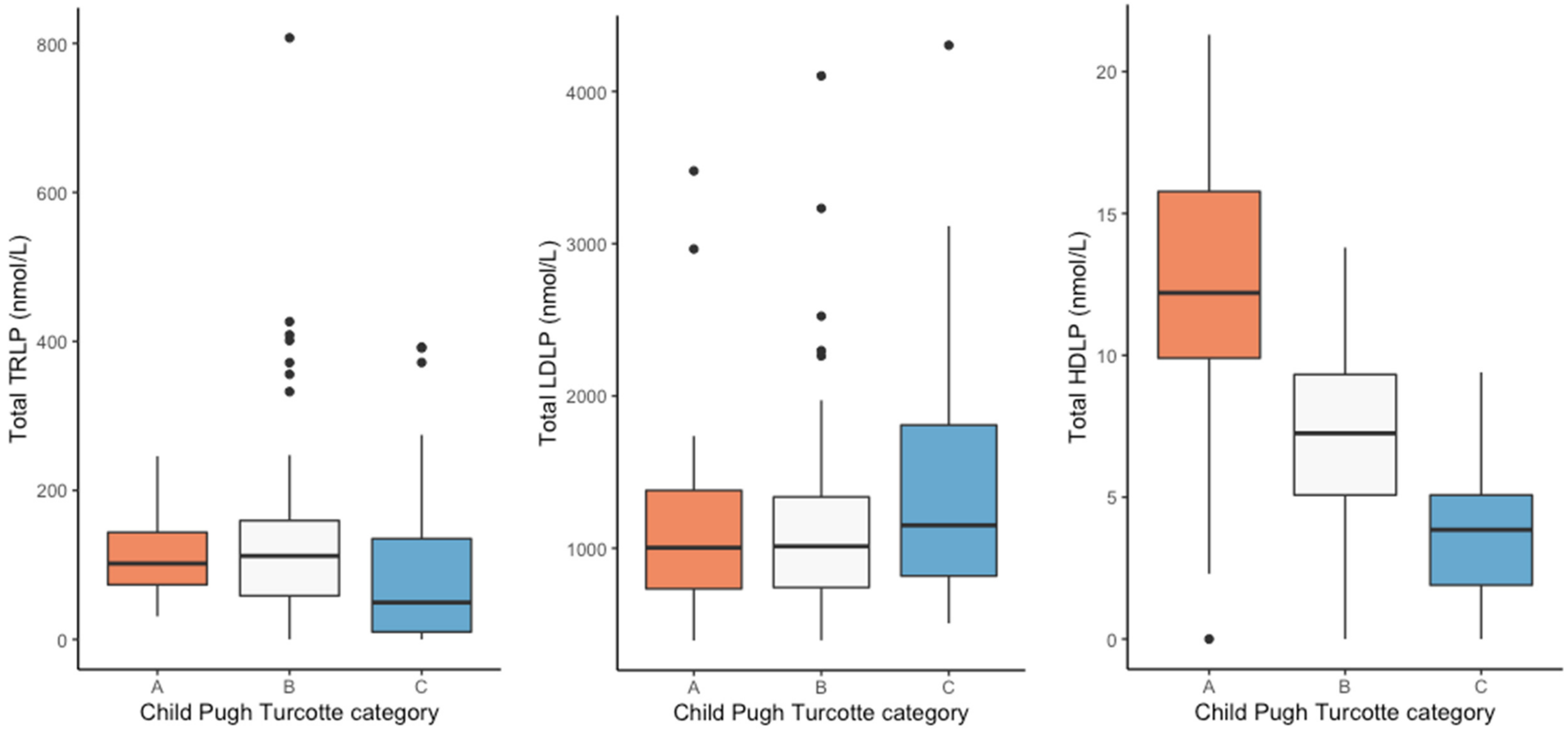

P-value: 0.026 *P*-value: 0.248 *P*-value: <0.001

Supplemental Figure S1. Boxplot of TRLP, LDLP and HDLP according to Child Pugh Turcotte classification in pre-transplant cirrhotic group. Boxplot A: TRLP, Boxplot B: LDLP, Boxplot C: HDLP. *P*-values were calculated with Kruskal Wallis Test. Abbreviations: HDLP, high density lipoprotein particles; LDLP, low density lipoprotein particles; LP-Z, lipoprotein Z; TRLP, triglyceride-rich lipoprotein particles.

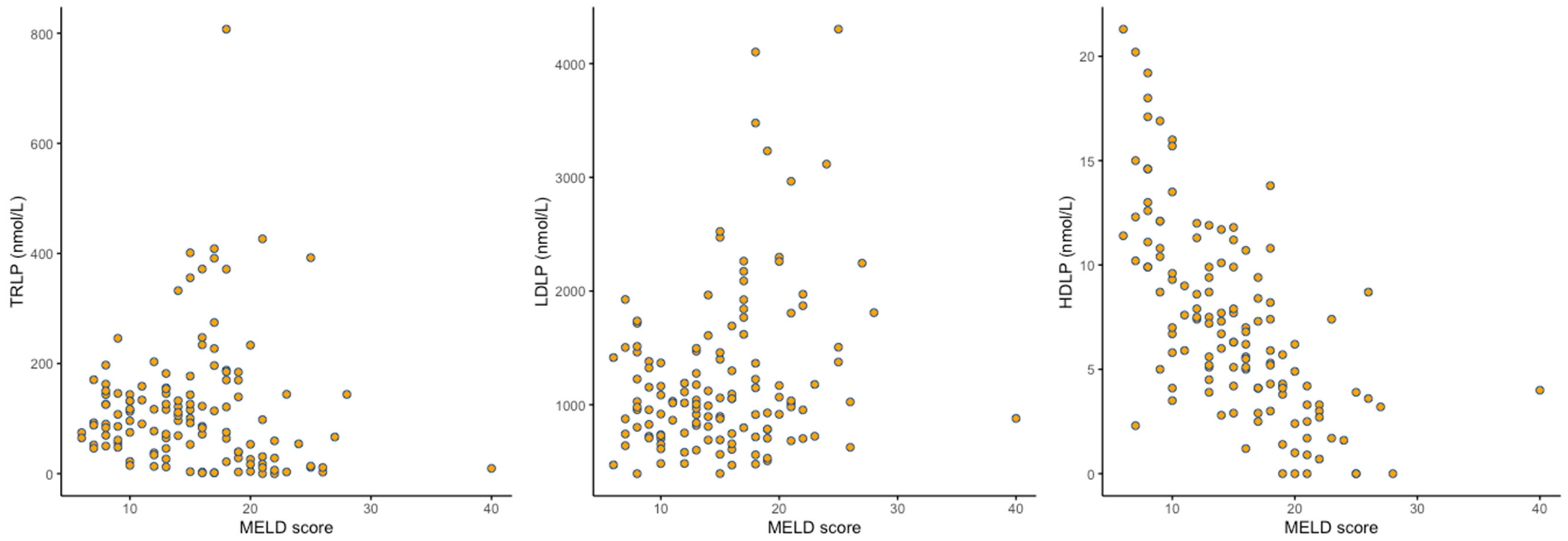

A. Correlation coefficient -0.178
P-value: 0.042

B. Correlation coefficient 0.190
P-value: 0.030

C. Correlation coefficient -0.712,
P-value: <0.001

Supplemental Figure S2. Distribution of TRLP, LDLP and HDLP according to MELD score in pre-transplant cirrhotic group. Figure A: TRLP, Figure B: LDLP, Figure C: HDLP. Spearman rank correlation coefficients with *P*-values are given. Abbreviations: HDLP, high density lipoprotein particle; LDLP, low density lipoprotein particles; LP-Z, lipoprotein Z; MELD score, model for end-stage liver disease score; TRLP, triglyceride-rich lipoprotein particles.
